# Safety and efficacy of thermal ablation for cervical metastatic lymph nodes in papillary thyroid carcinoma: A systematic review and meta-analysis

**DOI:** 10.3389/fendo.2022.967044

**Published:** 2022-08-22

**Authors:** Wanqing Tang, Xiuyun Tang, Danni Jiang, Xiaojuan Zhang, Rongling Wang, Xiaoyan Niu, Yichen Zang, Mingzhu Zhang, Xinya Wang, Cheng Zhao

**Affiliations:** ^1^ Department of Ultrasound, The Affiliated Hospital of Qingdao University, Qingdao, China; ^2^ Department of Ultrasound, Zibo Central Hospital, Zibo, China

**Keywords:** metastatic lymph nodes, thermal ablation, meta-analysis, systematic review, papillary thyroid carcinoma

## Abstract

**Background:**

To evaluate the safety and efficacy of radiofrequency ablation (RFA), microwave ablation (MWA), and laser ablation (LA) for the treatment of cervical metastatic lymph nodes (CMLNs) of papillary thyroid carcinoma (PTC).

**Methods:**

The Pubmed, EMBASE, Web of Science, and Cochrane Library databases were searched for studies on the safety and efficacy of thermal ablations (RFA, MWA, and LA) for the treatment of CMLNs of PTC until March 30, 2022. A review of 334 potential papers identified 17 eligible papers including 312 patients. Fixed-effects model or random-effects model was used to evaluate the pooled proportions of volume reduction rate (VRR), complete disappearance, and recurrence, and pooled estimates of changes in the largest diameter, volume, and serum *Tg* after ablation. The pooled proportions of overall and major complications were calculated. Subgroup analysis based on treatment modalities. The heterogeneity among studies was analyzed by using *Q* statistics and inconsistency index *I^2^*. MINORS scale was used to evaluate the quality of the studies.

**Results:**

17 eligible studies were finally identified, including 312 patients and 559 CMLNs. The pooled proportions of VRR, complete disappearance and recurrence of CMLNs were 91.28% [95% confidence interval *(CI)*: 86.60-95.97%], 67.9% [95% *CI*: 53.1-81.1%] and 7.8% [95%*CI*: 3.0-14.1%], respectively. The pooled estimates of changes in the largest diameter, volume and serum *Tg* were 8.12 mm [95%*CI*: 6.78-9.46 mm], 338.75 mm^3^ [95%*CI*: 206.85 -470.65 mm^3^] and 5.96 ng/ml [95%*CI*: 3.68-8.24 ng/ml], respectively. The pooled proportions of overall and major complications were 2.9% [95%*CI*: 0.3-7.1%] and 0.3% [95%*CI*: 0-1.9%], respectively. Significant between-study heterogeneity was observed for complete disappearance (*P*<0.01, *I^2^ =*88.6%), VRR (*P*<0.001, *I^2^ =*99.9%), recurrence (*P*=0.02, *I^2^ =*47.76%), overall complications (*P*<0.02, *I^2^ =*44.8%), and changes in the largest diameter (*P* < 0.001, *I^2^ =*82.6%), volume (*P*<0.001, *I^2^ =*97.0%), and serum *Tg* (*P* < 0.001, *I^2^ =*93.7%). Subgroup analysis showed heterogeneity of the VRR among the treatment modality (*I^2^* range: 84.4-100%). The VRR of MWA was the highest (97.97%), followed by RFA (95.57%) and LA (84.46%) (*P* < 0.001).

**Conclusion:**

All thermal ablations were safe and effective for the treatment of CMLNs of PTC. However, each treatment had significant heterogeneity in VRR. Compared with RFA and MWA, LA was less effective in reducing the volume of CMLNs of PTC.

## Introduction

Papillary thyroid carcinoma (PTC) is the most common pathological type of thyroid carcinoma, accounting for about 80-90% of thyroid malignant tumors (1). Thyroidectomy and radioactive iodine therapy are the main methods for the treatment of PTC, but 20-30% of patients have recurrence and lymph node metastasis after operation (2). Although reoperation is still the main treatment for cervical metastatic lymph nodes (CMLNs) of PTC (3, 4), the difficulty of reoperation and the risk of postoperative complications are increased due to the neck distortion, scar formation, and fibrous tissue adhesion (5, 6). Reoperation is not suitable for patients with high surgical risk or patients who refuse surgery. The guidelines of the American Thyroid Association recommend active surveillance for small CMLNs (7). However, some patients will have a sudden increase in the volume or number of CMLNs during the follow-up, and some patients will seek intervention due to psychological pressure.

In recent years, thermal ablation has developed rapidly and has been widely used for the treatment of benign (8–10) and malignant (11–13) thyroid nodules. Its application in CMLNs of PTC is also increasing and the therapeutic effect is remarkable (14). More and more evidence indicates that thermal ablations, including radiofrequency ablation (RFA), microwave ablation (MWA), and laser ablation (LA), are safe and effective for the treatment of CMLNs. However, most of the studies are retrospective, the sample size is small, and there is a lack of comparative study of various thermal ablation methods. Therefore, it is still necessary to comprehensively evaluate the efficacy and safety of thermal ablations for CMLNs of PTC. Therefore, this study aims to evaluate the safety and efficacy of thermal ablation for the treatment of CMLNs of PTC.

## Materials and methods

### Literature search strategy

This meta-analysis was performed according to the Preferred Reporting Items for a Systematic Review and Meta-analysis (PRISMA) guidelines (15). The Pubmed, EMBASE, Web of Science, and Cochrane Library databases were searched to identify publications on thermal ablation in treating CMLNs of PTC. The beginning date of literature was not limited, and the search was finalized on March 30, 2022. The search terms used were as follows: [(thyroid) AND (carcinoma OR cancer) AND (radiofrequency ablation OR RFA OR laser ablation OR LA OR microwave ablation OR MWA OR thermal ablation) AND (lymph node OR metastasis OR metastatic lymph node)]. References for selected articles were also further reviewed to find relevant articles. Two reviewers independently conducted the literature search, and any disagreement in the inclusion of articles was resolved by discussion until consensus was reached. We were not blinded to authors, institutions, journals, or interventions while selecting studies or extracting data.

### Inclusion and exclusion criteria

Studies investigating thermal ablations (i.e., RFA, LA, and MWA) for the treatment of CMLNs of PTC were eligible for inclusion. The included studies met the following criteria: 1) Population and intervention: patients were diagnosed with CMLNs of PTC; the main treatment was either RFA, LA, or MWA; and sample size ≥ 5 patients; 2) Study design: both retrospective and prospective studies were included; 3) Outcomes: the therapeutic effect (i.e., volume reduction, complete disappearance, recurrence, and complications) was reported in detail.

The exclusion criteria included the following: 1) case reports or series with< 5 patients; 2) abstracts, reviews, comments, and editorials; 3) articles not written in English. Two reviewers independently reviewed the literature in consensus.

### Data extraction

We extracted the following data from the included studies into standardized formats: (a) study characteristics: first author, year of publication, affiliation, duration of study, study design, and sample size; (b) patients’ demographic and clinical characteristics: age, sex, ablation techniques (RFA, LA or MWA), nodule characteristics (largest diameter and volume), and follow-up time; (c) outcomes: changes in the largest diameter (mm), serum *Tg* (ng/ml), and volume (mm^3^); volume reduction rate(VRR) (%) calculated as [(initial volume - final volume)/initial volume x 100%], complete disappearance rate [determined by ultrasound at final follow-up], and recurrence; (d) safety: frequency of overall and major complications. One reviewer extracted the data, and the other confirmed the validity of the data.

### Quality assessment

The quality of the included studies was independently assessed then in consensus by two reviewers using the structured criteria of MINORS scale.

### Definitions of complications

The Society of Interventional Radiology defined the complications caused by thermal ablation, including major and minor complications (16, 17). A major complication was defined as one that might be life-threatening, lead to substantial morbidity or disability, or prolong hospitalization time if left untreated. In this study, major complications included hypothyroidism, transient (lasting> 1 month) or permanent hoarseness. Minor complications included hoarseness (lasting < 1 month), hematoma, and pain requiring medication. Side effects were symptoms that did not require treatment or medication, including mild, transient postoperative pain, heat sensation, fever, local infection, and neck discomfort.

### Data synthesis and statistical analysis

The primary outcomes of this meta-analysis were the pooled proportions of VRR, complete disappearance, recurrence, overall complications, and major complications, and pooled estimates of changes in the largest diameter, volume, and serum *Tg*. In the meta-analysis, the weighted mean difference (WMD) and 95% confidence interval (CI) of continuous variables were evaluated, and the pooled proportions and 95%*CI* of categorical variables were evaluated. We used the inconsistency index *I^2^* with the *Q* statistic and *P*-value to evaluate the heterogeneity (18, 19). When *I^2^* >50% or *P*<0.05, we chose the random-effects model; in contrast, we used the fixed-effects model when *I^2^* <50% or *P*>0.05 *(* 19, 20). At the same time, subgroup meta-analysis was performed by treatment modality (RFA, LA, and MWA). Funnel plots and Egger’s test were used to evaluate publication bias, *P*<0.05 indicated significant publication bias (21). Sensitivity analysis was performed to identify the influence of an individual study on pooled estimates by removing one study at a time (18). All data were analyzed using STATA software version 15.0 (StataCorp, College Station, TX).

## Results

### Literature search

**Figure 1:**
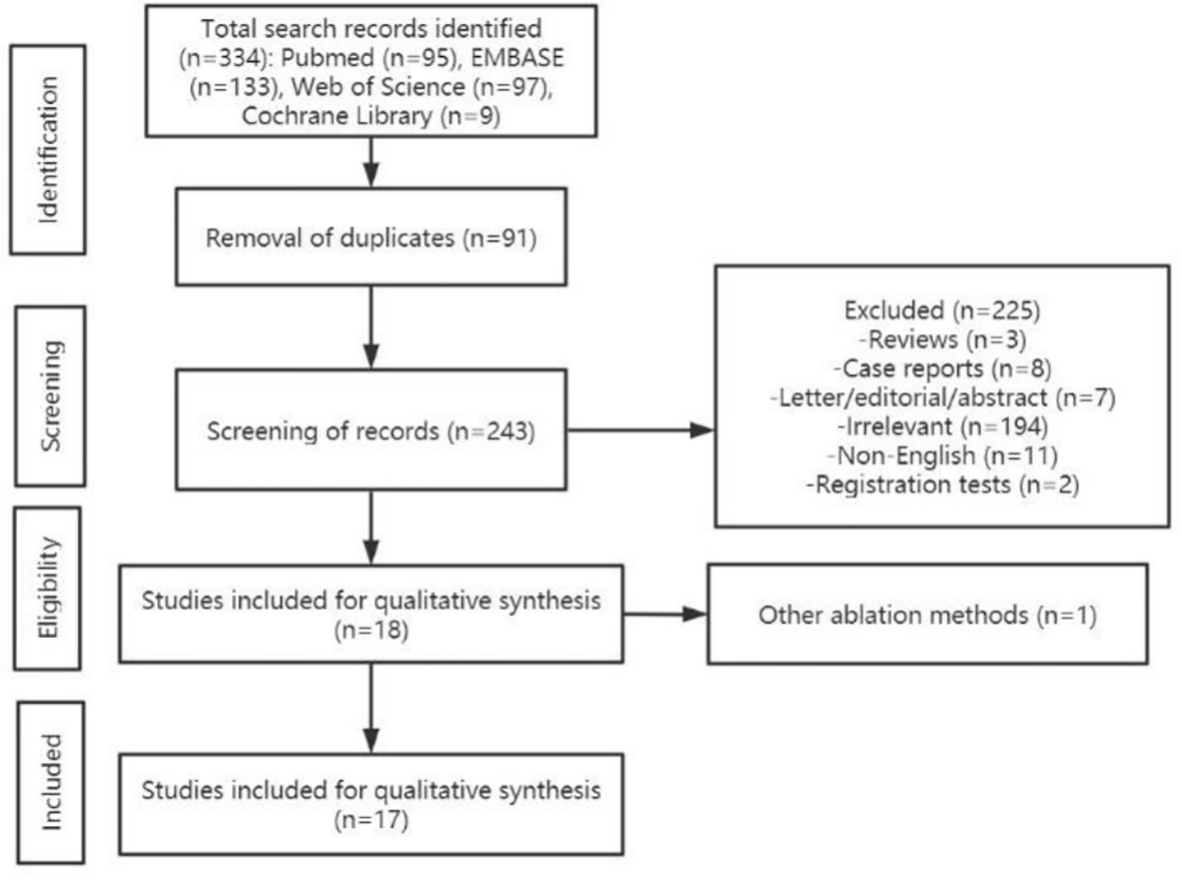
Flow diagram of the study selection process.

The study selection procedure was displayed in **Figure 1**. The initial literature search identified 334 articles. After removal of duplicates, 243 were screened for eligibility. Among them, 226 were excluded after reviewing the abstracts (3 review articles, 8 case reports, 7 letters/editorial/abstract, 194 articles of irrelevant contents, 1 article with other ablation methods, 2 registration tests, and 11 non-English articles). Then the full texts of the remaining 17 articles were thoroughly reviewed. Finally, 17 articles with a total of 312 patients with 559 CMLNs of PTC were included (22–38). After searching the bibliography of these articles, no additional eligible study was identified.

### Characteristics of included studies

The detailed characteristics of the 17 eligible studies are shown in **Table 1**. Among the 17 studies included, there were 13 retrospective studies (22–24, 26–32, 34, 36, 37) and 4 prospective studies (25, 33, 35, 38). All included studies were conducted in single centers. The cohort sizes varied from 5 to 45 patients with numbers of CMLNs ranging from 8 to 98. The mean ages of included study’s patients ranged from 15.6 to 62.3 years. The mean follow-up period ranged from 6.0 to 52.0 months, with 13 studies reporting an average of more than one year (23–25, 27, 29–36, 38). The types of thermal ablation techniques included 5 MWA (11, 23–26), 6 RFA (27–32), and 6 LA (33–38). The institutions of 10 studies were in China (11, 23–26, 28, 30, 32, 36, 38), two studies were in South Korea (27, 31), and five studies were in Italy (29, 33–35, 37). The quality assessment by MINORS showed moderate overall quality of the included studies (**Table S1**). All studies clearly described the thermal ablation techniques and equipment (**Table 2**).

**Table 1:** Study characteristics.

Authors	Year	Location	Study design	period	No. of patients (Female/Male)	Age	No. of lymph nodes	Follow-up time (month)
Microwave ablation								
Han et al	2020	China	Retrospective	2015.6 - 2020.1	37 (20/17)	43.58 ± 13.77	98	11.09 ± 9.21
Zhou et al	2018	China	Retrospective	2017.1 - 2018.4	14 (11/3)	45.1 ± 12.1	21	8.4 ± 4.1
Teng et al	2018	China	Retrospective	2014.5 - 2015.6	11 (8/3)	40.36 ± 10.52	24	30.5 ± 8.7
Cao et al	2020	China	Retrospective	2015.11 - 2018.11	14 (11/3)	46.9 ± 11.9	38	23.6 ± 9.3
Yue et al	2015	China	Prospective	2010.10 - 2013.3	17 (14/3)	54.1 ± 13.6	23	13.1 ± 6.0
Radiofrequency ablation								
Bake et al	2011	Korea	Retrospective	2004.12 - 2018.6	10 (6/4)	43.2 ± 18.0	12	23.0 ± 5.5
Lim et al	2014	Korea	Retrospective	2008.9 - 2012.4	39 (25/14)	52.8 ± 16.7	61	26.4 ± 13.7
Wang et al	2014	China	Retrospective	2013.1 - 2014.8	8 (7/1)	43.6 ± 9.3	20	9.4 ± 5.1
Yan et al	2021	Italy	Retrospective	2014.12 - 2018.3	5 (3/2)	15.6 ± 2.97	10	52.00 ± 21.44
Guang et al	2017	China	Retrospective	2013.7 - 2014.8	33 (22/11)	43.7 ± 10.7	54	21 ± 4
Guang et al	2018	China	Retrospective	2013.7 - 2014.12	45 (29/16)	41.5 ± 11.4	71	23 ± 5
Laser ablation								
Offi et al	2021	Italy	Retrospective	2019.6 - 2020.12	10 (5/5)	40.2 ± 17.98	10	6
Papini et al	2012	Italy	Prospective	2009.1 - 2010.12	5 (4/1)	53.6 ± 18.3	8	12
Mauri et al	2016	Italy	Retrospective	2010.9 - 2013.12	24 (13/11)	62.3 ± 13.2	46	30 ± 11
Mauri et al	2013	Italy	Prospective	2010.9 - 2012.4	15 (7/8)	62 ± 14	24	12
Guo et al.	2019	China	Retrospective	2016.6 - 2017.9	8 (5/3)	39.0 ± 11.6	18	12.8 ± 2.1
Zhang et al	2017	China	Prospective	2014.1 - 2015.3	17 (14/3)	43.94 ± 10.5	21	17.86 ± 4.70

**Table 2:** Details of ablation procedure.

Authors	Year	Power (W)		Ablation time (s)		Energy (J)	
		Mean ± SD	Range	Mean ± SD/median (interquartile range)	Range	Mean ± SD	Range
Microwave ablation							
Han et al	2020	20	NA	206.55 ± 193.59	NA	NA	NA
Zhou et al	2018	36.4 ± 2.3	35 - 40	93.9 ± 56.9	30 - 190	NA	NA
Teng et al	2018	20	NA	75.63 ± 45.44	12 - 174	1512.50 ± 908.77	240 - 3480
Cao et al	2020	30	NA	29 (20 - 49)	10 - 685	NA	NA
Yue et al	2015	40	NA	NA	NA	NA	NA
Radiofrequency ablation							
Bake et al	2011	14.7 ± 8.7	10 - 40	402.5 ± 325.2	60 - 900	6167.5 ± 7723.1	600 - 28800
Lim et al	2014	21 ± 6.9	10 - 50	243.5 ± 264.7	33 - 1200	3936.4 ± 5960.9	370 - 36000
Wang et al	2014	30	20 - 35	162 ± NA	30 - 360	NA	NA
Yan et al	2021	3.6 ± 1.3	3 - 6	158.80 ± 65.13	98 - 263	522.00 ± 211.59	290 - 760
Guang et al	2017	3.1 ± 0.5	3 - 5	140.7 ± 88.4	25 - 447	426.7 ± 279.8	70 - 1320
Guang et al	2018	3.3 ± 0.7	3 - 5	153.6 ± 91.7	25 - 479	439.2 ± 287.5	70 - 1450
Laser ablation							
Offi et al	2021	3.1 ± 0.3	3 - 4	531.86 ± 109.5	270 - 420	1256 ± 396	810 - 2720
Papini et al	2012	3	NA	NA	NA	942 ± 342	573 - 1574
Mauri et al	2016	NA	3 - 4	NA	NA	NA	1200 - 4200
Mauri et al	2013	3	NA	NA	NA	NA	1200 - 4200
Guo et al.	2019	3.2 ± 0.2	3 - 3.5	NA	NA	1.4 ± 0.3	1 - 1.8
Zhang et al	2017	NA	NA	NA	NA	NA	NA

NA, not applicable.

### Changes in the largest diameter after MWA, RFA and LA

**Figure 2:**
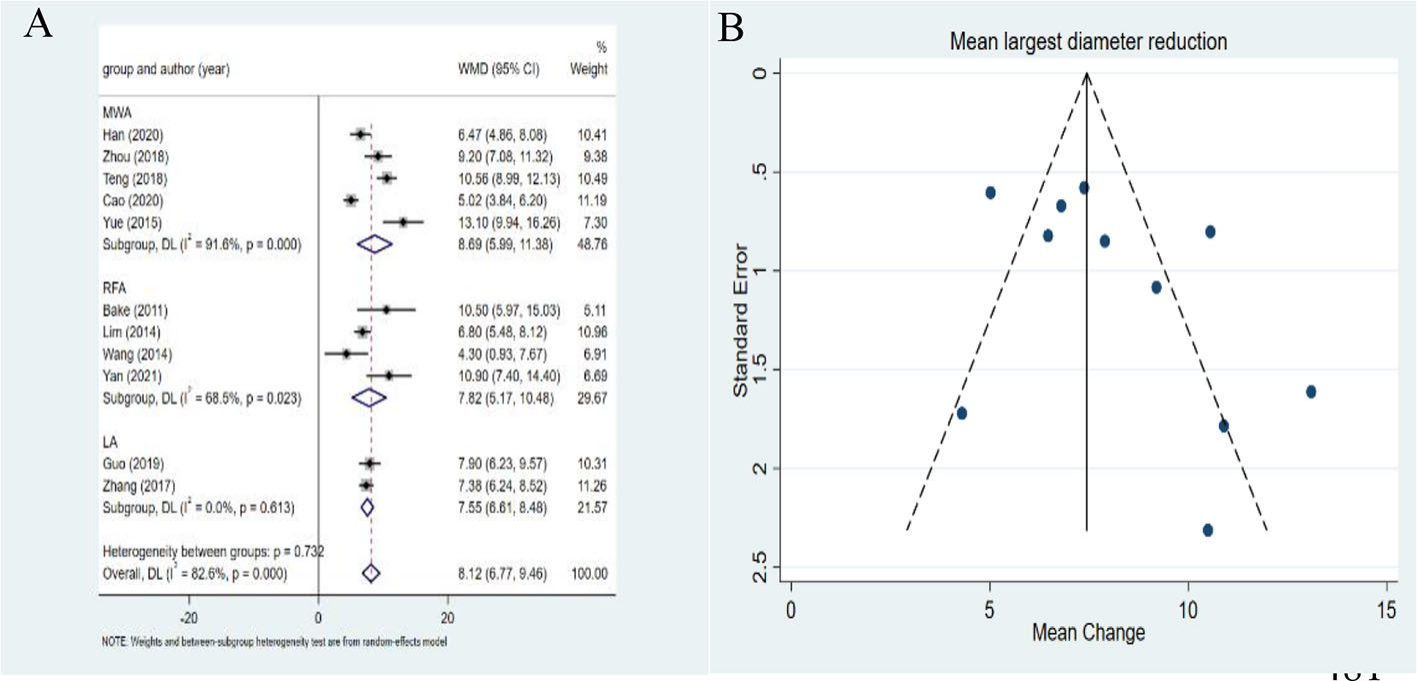
Forest plots **(A)** and funnel plots **(B)** of reduction of largest diameter of metastatic lymph nodes.

The overall pooled estimates for the WMD in largest diameter from baseline to the last follow-up after ablation are summarized in **Figure 2** with corresponding forest plots. The heterogeneity among studies was significant (*I^2^* = 82.6%, *P* < 0.001), so the random-effects model was used for the analysis. The pooled WMD was 8.12mm (95%*CI*: 6.77-9.46mm). RFA, LA, and MWA all induced a statistically significant reduction in largest diameter after ablation (all *P*<0.001). In addition, MWA achieved a higher pooled WMD (8.69mm, 95%*CI*: 5.99-11.38mm) than RFA (7.82mm, 95%*CI*: 5.17-10.48mm) and LA (7.55mm, 95%*CI*: 6.61-8.48mm). The differences in the pooled WMD among the three therapies in the largest diameter were statistically significant (*P <*0.001). No publication bias was found in funnel plot and Egger’s test (*P*=0.132).

### Changes in the volume after MWA, RFA and LA

**Figure 3:**
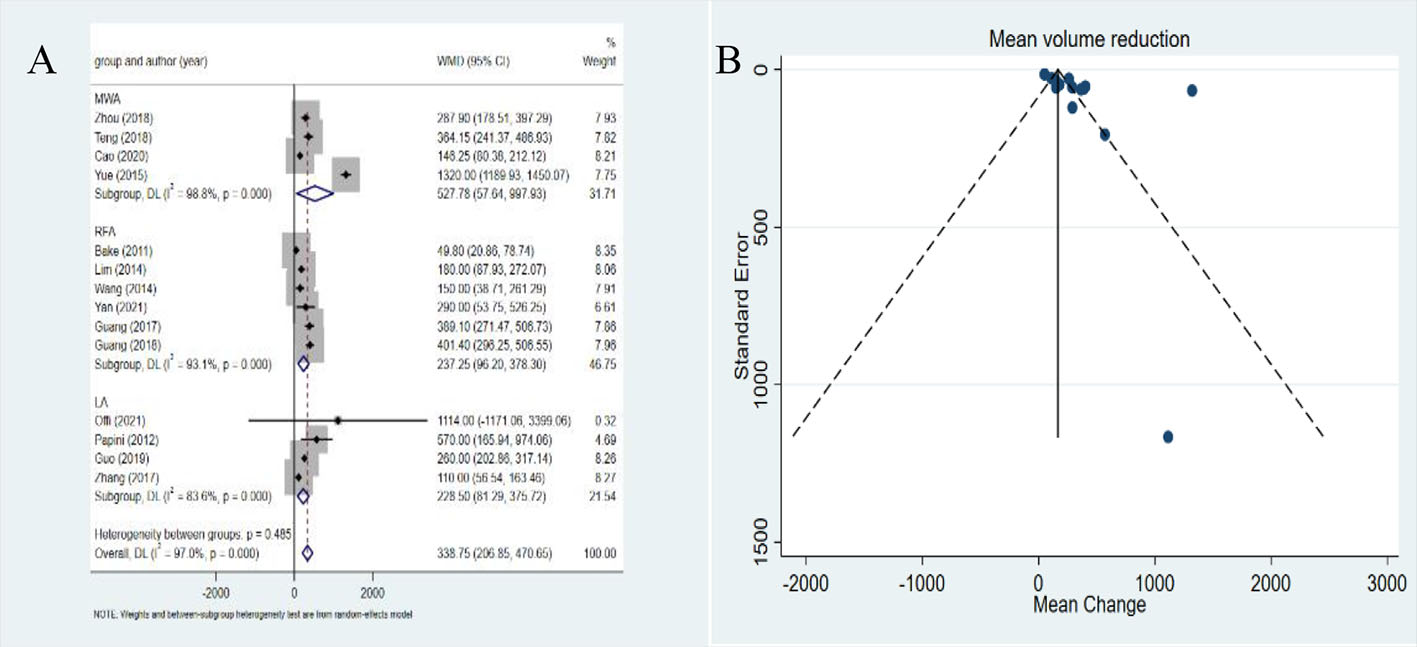
Forest plots **(A)** and funnel plots **(B)** of reduction of volume of metastatic lymph nodes.

The overall pooled estimates for the WMD in tumor volume from baseline to the last follow-up after ablation are summarized in **Figure 3** with corresponding forest plots. The pooled WMD was 338.75mm^3^ (95%*CI*: 206.85-470.65mm^3^). A significant heterogeneity was observed (*I^2^ =*97.0%, *P*<0.001). RFA, LA, and MWA all induced a statistically significant reduction in tumor volume after ablation (*P*=0.001, *P*=0.002, *P*=0.028 in the RFA, LA and MWA groups, respectively). In addition, MWA achieved a higher pooled WMD (527.78mm^3^,95%*CI*: 57.64-997.93mm^3^) than RFA (237.25mm^3^,95%*CI*: 96.20-378.30mm^3^) and LA (228.50mm^3^,95%*CI*: 81.29-375.72mm^3^). The differences in the pooled WMD among the three therapies were statistically significant (*P*<0.001). The funnel plot and Egger’s test showed a significant publication bias (*P*=0.025). After the trim-and-fill method adjusting publication bias, the adjusted pooled estimates for mean volume reduction were still 338.75mm^3^ (95%*CI*: 206.85-470.65mm^3^).

### Pooled estimates of mean volume reduction rate

**Figure 4:**
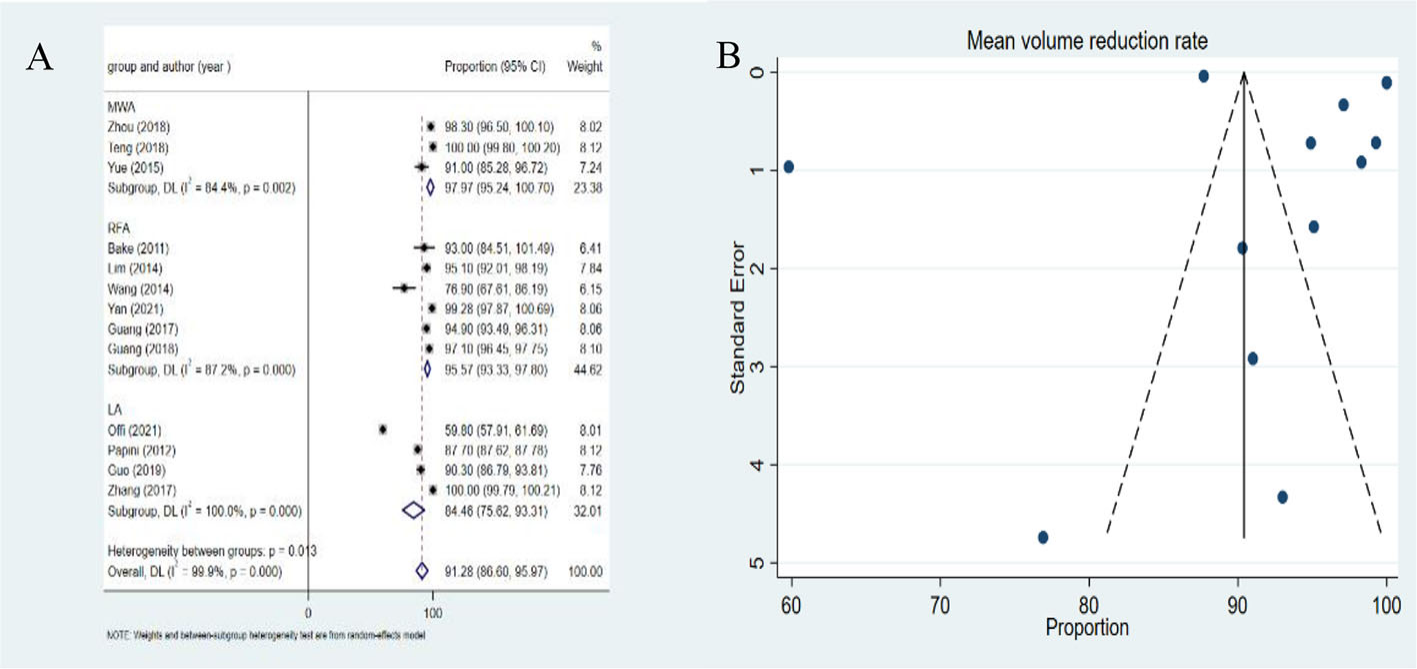
Forest plots **(A)** and funnel plots **(B)** of volume reduction rate of metastatic lymph nodes.

The pooled estimate of VRR was 91.28% (95%*CI*: 86.60-95.97%) (**Figure 4A**). There was significant heterogeneity for VRR (*I^2^ =*99.9%, *P*<0.001). MWA achieved a higher pooled proportion (97.97%, 95%*CI*: 95.24-100.70%) than RFA (95.57%,95%*CI*: 93.33-97.80%) and LA (84.46%, 95%*CI*: 75.62-93.31%). The differences in the pooled proportion among the three modalities were statistically significant (*P*<0.001). No publication bias was found in funnel plot and Egger’s test (*P*=0.364) (**Figure 4B**).

### Changes in the serum Tg after MWA, RFA and LA

**Figure 5:**
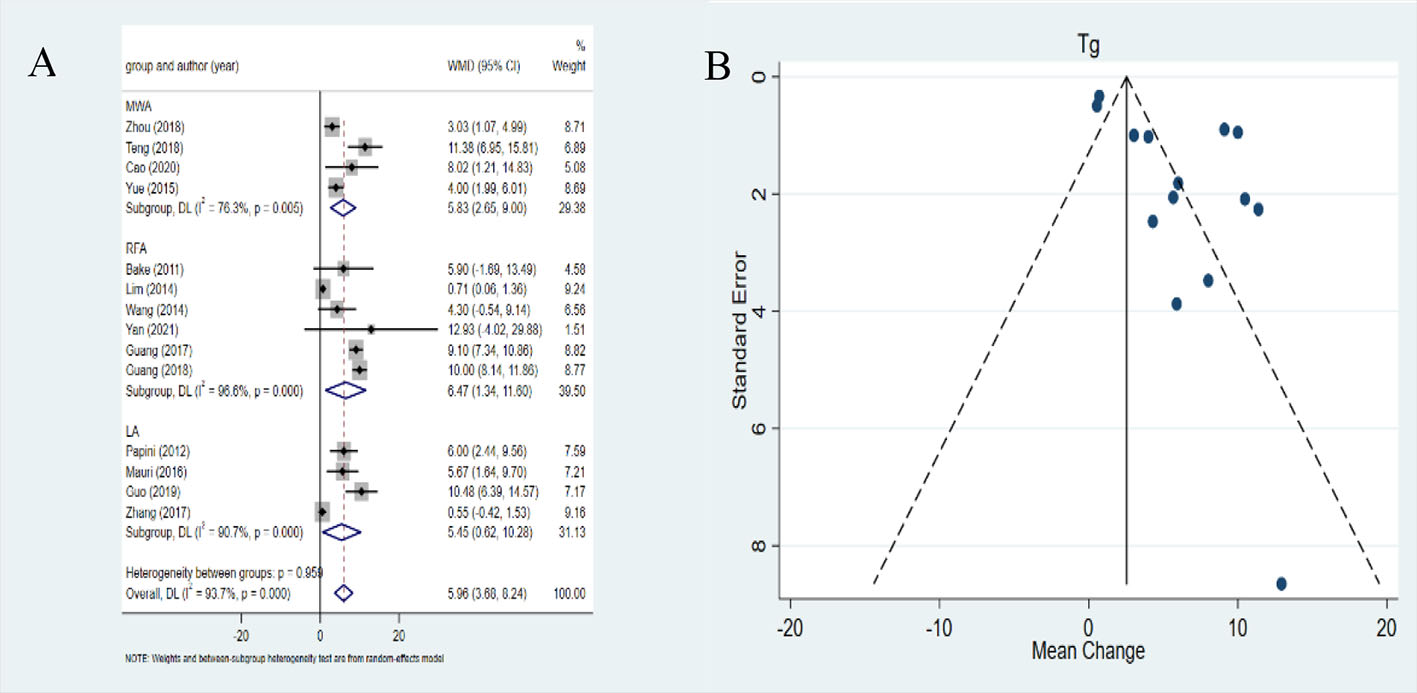
Forest plots **(A)** and funnel plots **(B)** of change of thyroglobulin levels.

The pooled WMD was 5.96ng/ml (95%*CI*: 3.68-8.24ng/ml) (**Figure 5A**). A significant heterogeneity was noted in the serum Tg (*I2 =*93.7%, *P*<0.001). RFA, LA, and MWA all induced a statistically significant reduction in serum *Tg* after ablation (*P*= 0.013, *P*=0.027, and *P*<0.001 in the RFA, LA, and MWA groups, respectively). In addition, RFA yielded higher pooled WMD (6.47 ng/ml, 95%*CI*: 1.34-11.60 ng/ml) than LA (5.45 ng/ml, 95%*CI*: 0.62-10.28 ng/ml) and MWA (5.83 ng/ml, 95%*CI*: 2.65-9.00 ng/ml). The differences in the pooled WMD among the three therapies in serum *Tg* were statistically significant (*P*<0.001). The funnel plot and Egger’s test showed a significant publication bias (*P*=0.011) (**Figure 5B**). After the trim-and-fill method adjusting publication bias, the adjusted pooled estimates for serum *Tg* were 5.436 ng/ml (95%*CI*: 3.264-7.609 ng/ml).

### Pooled proportions of complete disappearance

**Figure 6:**
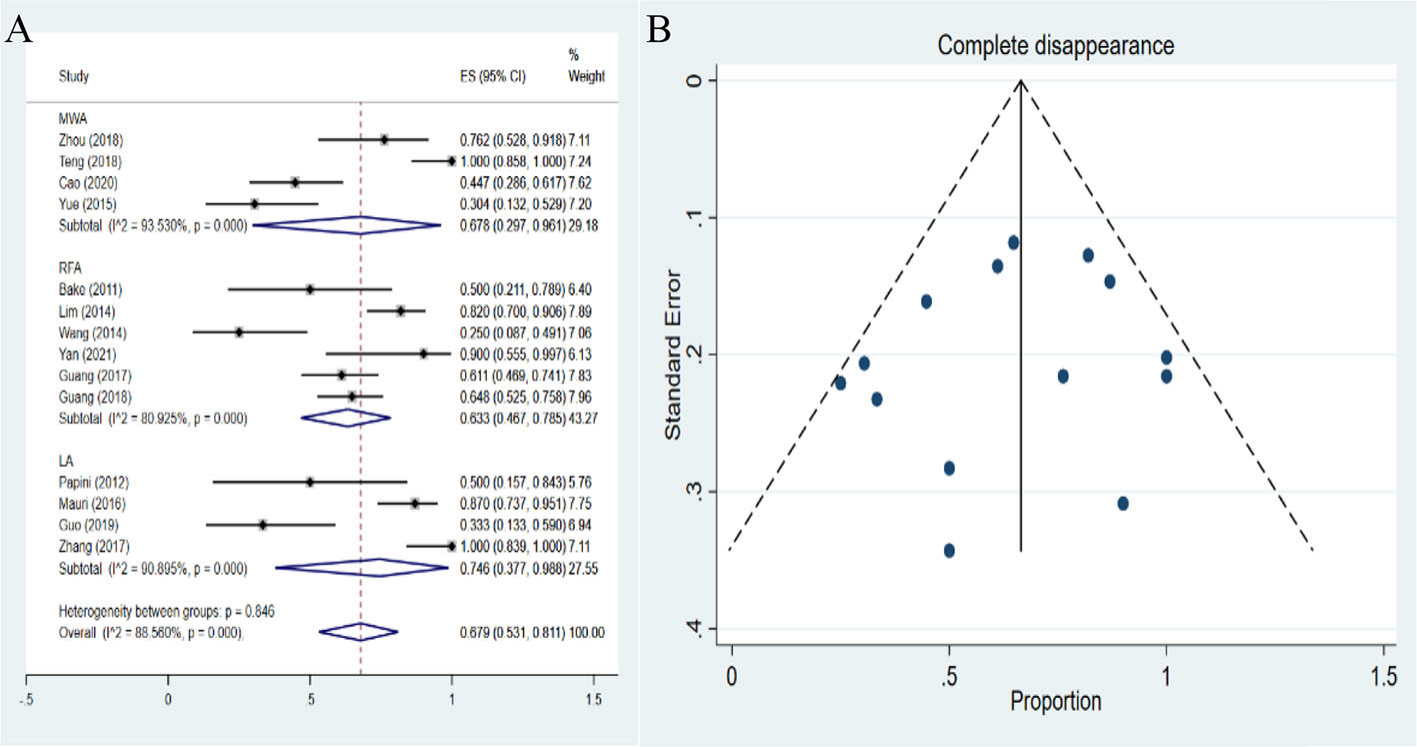
Forest plots **(A)** and funnel plots **(B)** of pooled complete disappearance.

The complete disappearance demonstrated a pooled proportion of 63.3% (95%*CI*: 46.7-78.5%), 74.6% (95%*CI*: 37.7-98.8%) and 67.8% (95%*CI*: 29.7-96.1%) after RFA, LA and MWA treatment, respectively (**Figure 6A**). The heterogeneity among studies was significant (*I^2^ =*88.56%, *P*<0.01). These results showed that the proportion of complete disappearance after LA was higher than the other two therapies. The differences in the pooled proportion among the three therapies in complete disappearance were statically significant (*P*<0.001). No publication bias was found in funnel plot and Egger’s test (*P*=0.523) (**Figure 6B**).

### Pooled proportions of recurrence

**Figure 7:**
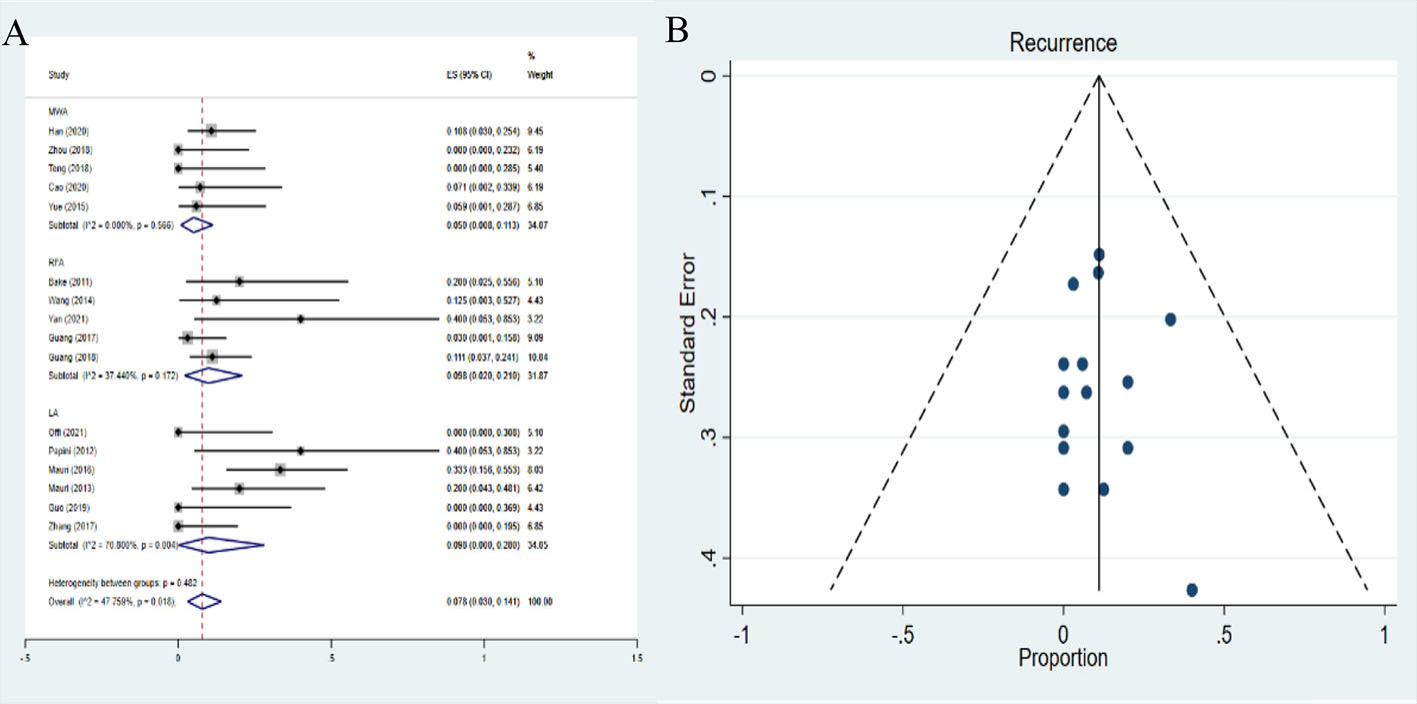
Forest plots **(A)** and funnel plots **(B)** of pooled recurrence.

The recurrence demonstrated a pooled proportion of 9.8% (95%*CI*: 2.0-21.0%), 9.8% (95%*CI*: 0-28.0%), and 5.0% (95%*CI*: 0.8-11.3%) after RFA, LA and MWA treatment, respectively (**Figure 7A**). The results showed that the proportion of recurrence after MWA was lower than the other two therapies. The differences in the pooled proportion among the three therapies in recurrence were statically significant (*P*<0.01). No publication bias was found in funnel plot and Egger’s test (*P*=0.533) (**Figure 7B**).

### Pooled proportions of complications

**Figure 8:**
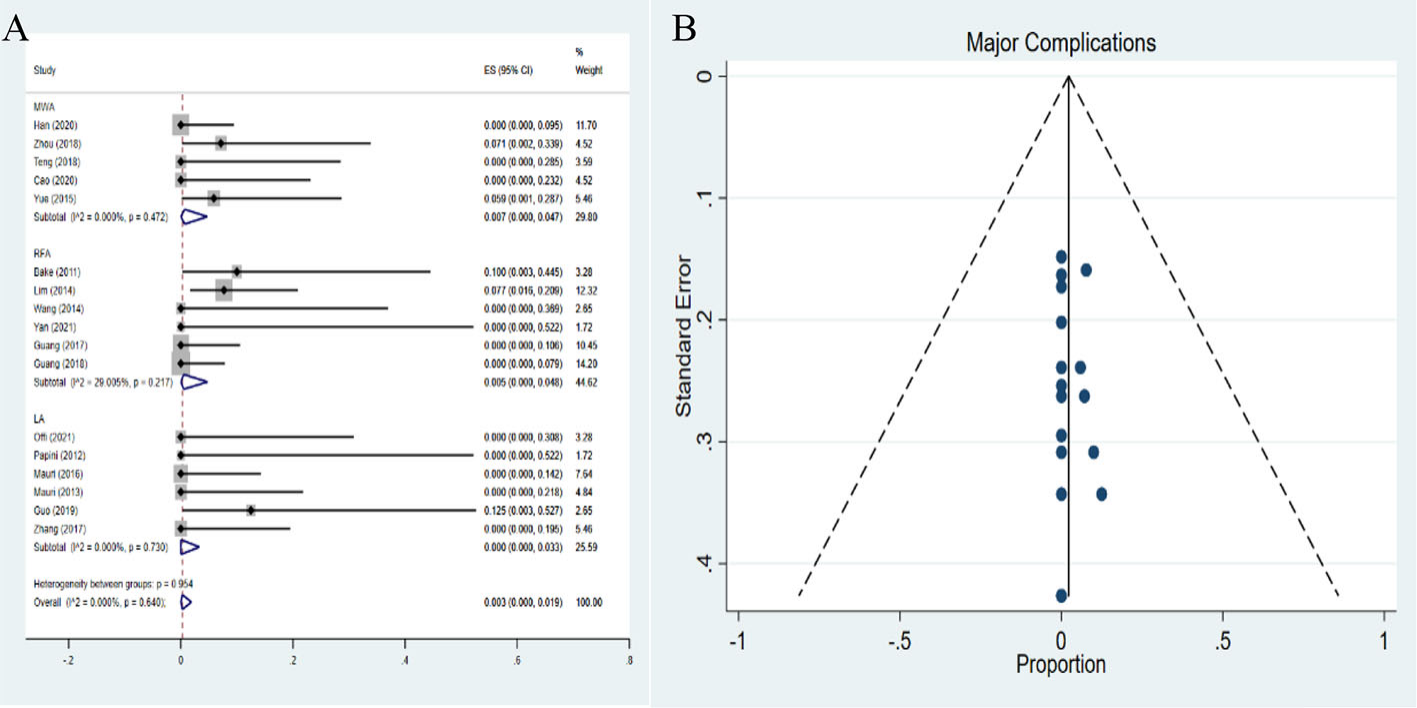
Forest plots **(A)** and funnel plots **(B)** of pooled major complications.

**Figure 9:**
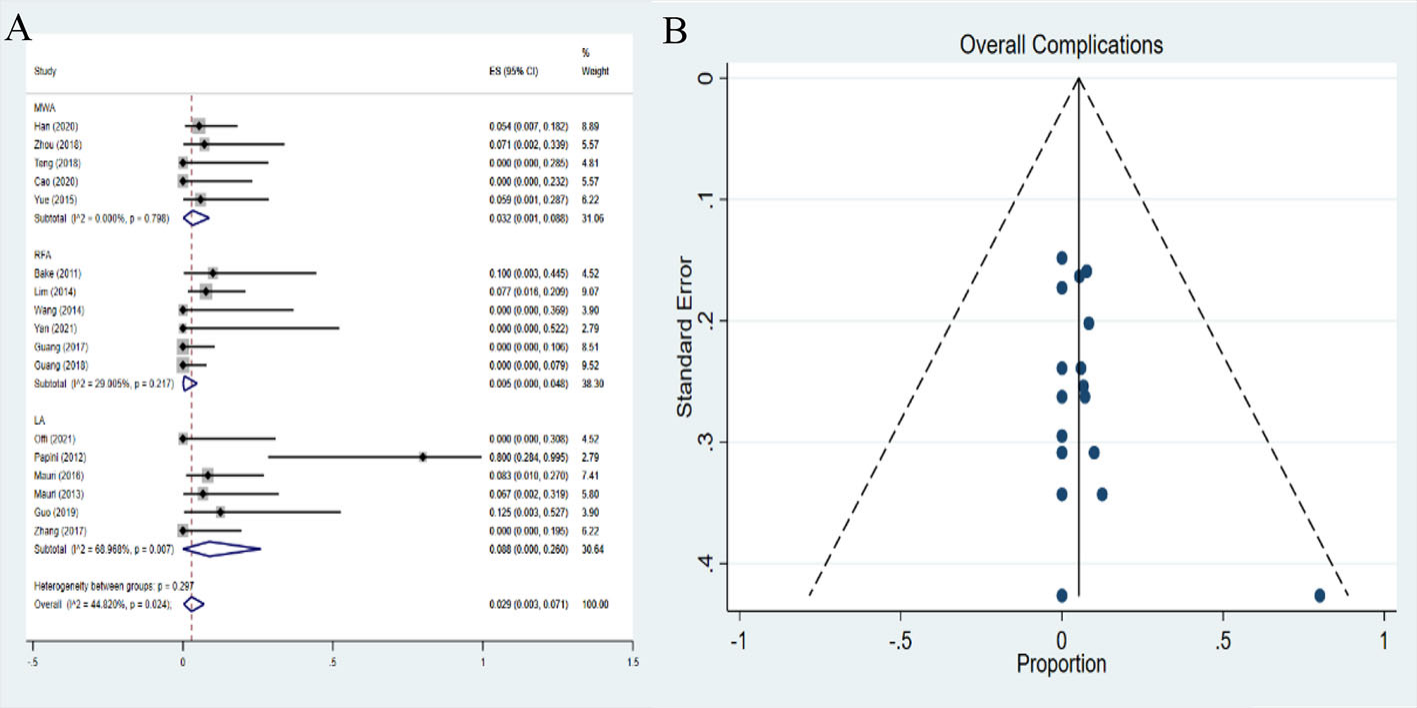
Forest plots **(A)** and funnel plots **(B)** of pooled overall complications.

16 complications among 559 CMLNs in 312 patients were reported. The pooled proportion of major complications was 0.3% (95% *CI*: 0-1.9%). The major complications demonstrated pooled proportions of 0.7% (95%*CI*: 0-4.7%), 0.5% (95%*CI*: 0-4.8%), and 0% (95%*CI*: 0-3.3%) in MWA, RFA and LA, respectively (**Figure 8A**). However, no statistical significance was observed among the three therapies (*P*=0.332). No publication bias was seen for major complications (*P*=0.622) (**Figure 8B**). The pooled proportion of overall complications was 2.9% (95%*CI*: 0.3-7.1%). The pooled proportions of overall complications were 3.2% (95%*CI*: 0.1 -8.8%), 0.5% (95%*CI*: 0- 4.8%), and 8.8% (95%*CI*: 0 -26.0%) in MWA, RFA and LA, respectively (**Figure 9A**). These results demonstrated that the overall complications of RFA was the lowest. The differences in the pooled proportion among the three therapies in overall complications were statistically significant (*P*=0.008). No publication bias was found in funnel plot and Egger’s test (*P*=0.136) (**Figure 9B**).

### Details on major and minor complications

All seven major complications were voice changes lasting at least one month. Nine minor complications were reported including 2 cases of skin scald, 1 case of hematoma, 3 cases of pain, and 3 cases of hoarseness lasting less than 1 month. All hoarseness resolved within 1 month after ablation. In addition to pain relieved by medication, all minor complications resolved spontaneously within 3 months. The incidences of major and minor complications were 2.2% (7/312) and 2.9% (9/312), respectively.

### Sensitivity analyses

A sensitivity analysis was used to evaluate the reliability of the results. When one study was deleted at a time, no such deletion had any significant impact on the results.

## Discussion

This meta-analysis showed that ultrasound-guided thermal ablation was an effective treatment for CMLNs of PTC. The pooled estimate for mean VRR of CMLNs after thermal ablation was 91.28% (95% *CI*: 86.60-95.97%), and the pooled estimate for serum *Tg* was 5.96 ng/ml (95%*CI*: 3.68-8.24 ng/ml). The pooled proportion of complete disappearance of CMLNs was 63.3% (95%*CI*: 46.7-78.5%), and the pooled proportion of recurrence after ablation was 7.8% (95%*CI*: 3.0 -14.1%). In addition, the pooled proportion of major complications was very low (0.3%, 95%*CI*: 0-1.9%), and all of them were non-life-threatening voice changes. Based on the above results, all three thermal ablation techniques have shown efficacy and safety for the treatment of CMLNs of PTC.

So far, many studies have described the safety and efficacy of thermal ablation for the treatment of CMLNs of PTC. However, there are no definitive guidelines on how RFA, LA, and MWA should be used to treat CMLNs of PTC, and few studies are comparing these three treatments (39, 40). In addition, due to the lack of well-designed prospective randomized trials, a direct comparison among them is difficult for the treatment of CMLNs of PTC. Therefore, our systematic review and meta-analysis collected the available evidence, which was crucial and will promote clinical practice.

Through subgroup analysis of treatment methods, the mean VRR of CMLNs was comparable between MWA (97.97%) and RFA (95.57%), while that of LA was lower (84.46%). This finding was consistent with a meta-analysis by Choi et al (41), who found comparable VRR between RFA and MWA in the treatment of papillary thyroid microcarcinoma. In addition, Javadov et al. (42) found that the VRR of RFA and MWA for the treatment of benign thyroid nodules was comparable, which was similar to our result. However, our findings were for CMLNs of PTC, rather than benign thyroid nodules and papillary thyroid microcarcinoma reported in these studies.

In our current study, we found that thermal ablation was effective in the treatment of CMLNs of PTC, although existing heterogeneity. It could be attributed to the following reasons. First, there were differences among the three thermal ablations. Second, the follow-up time of some studies was not long enough to observe the results, which may affect the accuracy of these results. Third, some studies reported that the proportion of complete disappearance was very low, which may be due to the residual calcification or scar-like lesions remained. These residues had not changed significantly after one year, but no obvious enhancement was observed on contrast-enhanced ultrasound, and no tumor cells were found in FNA. Lastly, the difference in recurrence may be related to different definitions of recurrence.

Overall, thermal ablation was safe for the treatment of CMLNs of PTC. The pooled proportions of overall (2.9%, 95%*CI*: 0.3-7.1%) and major (0.3%, 95% *CI*: 0 -1.9%) complications were similar to those previously reported in a meta-analysis evaluating the safety of thermal ablation for the treatment of papillary thyroid microcarcinoma (pooled proportions of overall and major complications:3.2% and 0.7%, respectively) (5). However, in a meta-analysis of the safety and efficacy of MWA for the treatment of benign thyroid nodules and papillary thyroid microcarcinoma, the pooled proportions of major and minor complications were 11.5% and 5.1% (43), respectively. All were higher than the results of this study. In fact, in the subgroup analysis by treatment modality, we found that the pooled proportion of major complications of MWA was higher than that of the other two methods, although the difference was not statistically significant. The reasons for this difference may be attributed to the following aspects. First of all, compared with other thermal ablations, MWA could achieve a larger ablation area in a shorter time (44, 45). The risk of thermal damage was greater, which was more likely to affect the surrounding recurrent laryngeal nerve, a relatively high incidence of major complications. Secondly, this difference may be related to variation in operators’ experience or procedural techniques, so it did not necessarily mean that the safety of MWA was lower than that of the other two methods. The pooled proportion of overall complications of RFA was lower than that of the other two treatments, and the difference was statistically significant. The low incidence of RFA complications may be due to the slow rise of RFA temperature and the easy removal of heat (46). Except for the slight difference in the incidence of complications among different treatments, no life-threatening complications were reported in all studies, which confirmed that all thermal ablations were safe.

In terms of heterogeneity, very little heterogeneity was present for the major complications of CMLNs of PTC (*P*=0.64, *I^2^ =*0%). However, significant between-study heterogeneity was noted for the largest diameter, volume, VRR, serum *Tg*, and complete disappearance of CMLNs of PTC (*P*<0.001). After subgroup analysis by treatment modality, we noticed that each treatment had great heterogeneity, with *I^2^* exceeding 80%. We believed that a small number of studies included in each subgroup might be attributable to this finding. In addition, the heterogeneity among studies was moderate in terms of recurrence and overall complications of CMLNs of PTC (*P*=0.02, *I^2^ =*47.76%; *P*=0.02, *I^2^ =*44.82%). LA accounted for most of the heterogeneity (*I^2^ =*70.80%; *I^2^ =*68.97%), while MWA showed no heterogeneity (*I^2^ =*0%), which may indicate that MWA may have more consistent treatment effects than LA.

Three treatment modalities are achieved by the thermal ablation mechanism (45). Compared with surgery, thermal ablation has the advantages of small trauma, low incidence of complications, and rapid recovery. It is a good treatment for patients with surgical contraindications or rejection of surgery. However, it should be noted that some small CMLNs may not be detected by ultrasound (47), which will reduce the therapeutic effect to some extent. In the included studies, the follow-up time varied from 6 to 52 months. For some studies, the follow-up time may not be long enough to accurately determine the efficacy of thermal ablations. Although some studies have not been included in our study due to the small sample size, it is undeniable that they have achieved good therapeutic effects. In the future, we need to further verify the efficacy and safety of thermal ablations in a longer follow-up period.

There are some limitations to our study. First, there are few studies included. Although thermal ablation develops rapidly, its application in CMLNs of PTC is less. Second, most studies are retrospective studies, which may affect the accuracy of the results. Moreover, the included studies were mainly concentrated in China, South Korea, and Italy. Our meta-analysis results may not be similar in other regions because of the different clinical practice environments in these regions.

In conclusion, the current systematic review and meta-analysis comprehensively reviewed the safety and efficacy of RFA, LA, and MWA for the treatment of CMLNs of PTC. Our results show that thermal ablation is safe and effective. However, future research needs longer follow-ups and more population studies.

## Data availability statement

The original contributions presented in the study are included in the article/**Supplementary Material**. Further inquiries can be directed to the corresponding author.

## Author contributions

WT, XT and DJ were major contributors in writing the manuscript. XZ, XN, and RW contributed to literature search, screening, and data extraction. YZ, MZ, and XW contributed to statistical analyses. WT and XT contributed to data validation. .WT and CZ are responsible for review and modification of the manuscript. All authors read and approved the final manuscript.

## Acknowledgments

All the authors of included original studies should be appreciated sincerely.

## Conflict of interest

The authors declare that the research was conducted in the absence of any commercial or financial relationships that could be construed as a potential conflict of interest.

## Publisher’s note

All claims expressed in this article are solely those of the authors and do not necessarily represent those of their affiliated organizations, or those of the publisher, the editors and the reviewers. Any product that may be evaluated in this article, or claim that may be made by its manufacturer, is not guaranteed or endorsed by the publisher.
