# Supplementary material for: Safety and efficacy of thermal ablation for cervical metastatic lymph nodes in papillary thyroid carcinoma: A systematic review and meta-analysis
Source: Front Endocrinol (Lausanne). 2022 Aug 22;13:967044. doi: 10.3389/fendo.2022.967044 (PMC9441577; doi:10.3389/fendo.2022.967044)
Supplement: Supplementary file 1 [file Table_1.docx]

Supplementary Material

# Supplementary Tables

Table S1. Quality assessment of non-RCT studies using the MINORS scale.

|  | **A clearly stated aim** | **Inclusion of consecutive patients** | **Prospective collection of data** | **Endpoints appropriate to the aim of the study** | **Unbiased assessment of the study endpoint** | **Follow-up period appropriate to the aim of the study** | **Loss to follow up less than 5%** | **Prospective calculation of the study size** | **Total quality scores** |  |
| --- | --- | --- | --- | --- | --- | --- | --- | --- | --- | --- |
| Han | 2 | 2 | 1 | 2 | 1 | 1 | 2 | 0 | 11 |  |
| Zhou | 2 | 2 | 1 | 2 | 1 | 1 | 2 | 0 | 11 |  |
| Teng | 2 | 2 | 1 | 2 | 1 | 2 | 2 | 0 | 12 |  |
| Cao | 2 | 2 | 1 | 2 | 1 | 1 | 2 | 0 | 11 |  |
| Yue | 2 | 2 | 2 | 2 | 1 | 1 | 2 | 0 | 12 |  |
| Bake | 2 | 1 | 1 | 2 | 1 | 1 | 2 | 0 | 10 |  |
| Lim | 2 | 2 | 1 | 2 | 1 | 1 | 2 | 0 | 11 |  |
| Wang | 2 | 1 | 1 | 2 | 1 | 1 | 2 | 0 | 10 |  |
| Yan | 2 | 2 | 1 | 2 | 1 | 1 | 2 | 0 | 11 |  |
| Guang | 2 | 2 | 1 | 2 | 1 | 1 | 2 | 0 | 11 |  |
| Guang | 2 | 1 | 1 | 2 | 1 | 1 | 2 | 0 | 10 |  |
| Offi | 2 | 1 | 1 | 2 | 1 | 1 | 2 | 0 | 10 |  |
| Papini | 2 | 2 | 2 | 2 | 1 | 1 | 2 | 0 | 12 |  |
| Mauri | 2 | 2 | 1 | 2 | 2 | 2 | 2 | 0 | 13 |  |
| Mauri | 2 | 1 | 2 | 2 | 1 | 1 | 2 | 0 | 11 |  |
| Guo | 2 | 1 | 1 | 2 | 2 | 1 | 2 | 0 | 11 |  |
| Zhang | 2 | 1 | 2 | 2 | 2 | 1 | 2 | 0 | 12 |  |

**
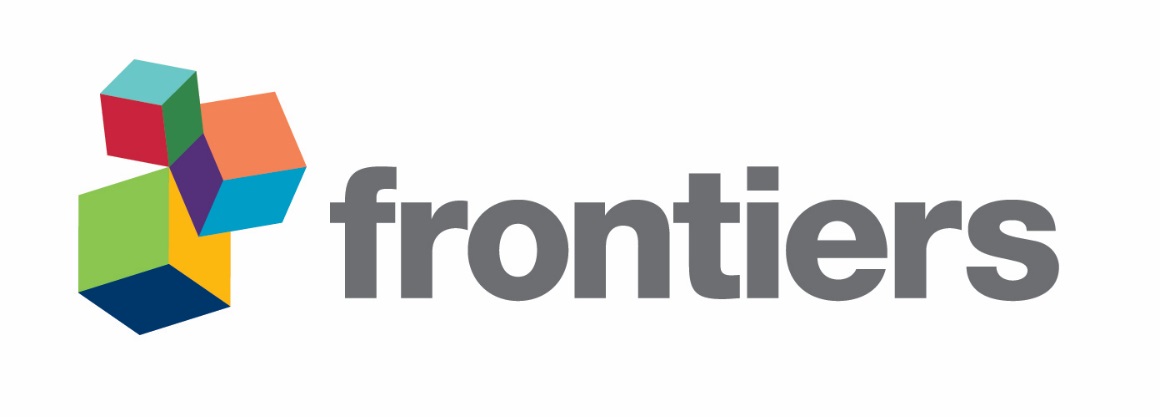
**
